# The comparative effects of manganese nanoparticles and their counterparts (bulk and ionic) in *Artemisia annua* plants *via* seed priming and foliar application

**DOI:** 10.3389/fpls.2022.1098772

**Published:** 2023-01-19

**Authors:** Hajar Salehi, Abdolkarim Cheheregani Rad, Ali Raza, Ivica Djalovic, P. V. Vara Prasad

**Affiliations:** ^1^ Laboratory of Plant Cell Biology, Department of Biology, Bu-Ali Sina University, Hamedan, Iran; ^2^ College of Agriculture, Fujian Agriculture and Forestry University, Fuzhou, China; ^3^ Institute of Field and Vegetable Crops, National Institute of the Republic of Serbia, Novi Sad, Serbia; ^4^ Department of Agronomy, Kansas State University, Manhattan, KS, United States

**Keywords:** antioxidant compounds, biostimulants, germination index, nanotechnology, nutripriming, seed priming

## Abstract

The world has experienced an unprecedented boom in nanotechnology. Nanoparticles (NPs) are likely to act as biostimulants in various plants due to having high surface/volume value. However, understanding the actual effect of NPs is essential to discriminate them from other counterparts in terms of being applicable, safe and cost-effective. This study aimed to assay the impact of manganese(III) oxide (Mn_2_O_3_)-NPs *via* seed-priming (SP) and a combination of SP and foliar application (SP+F) on *Artemisia. annua* performance at several times intervals and comparison with other available manganese (Mn) forms. Our findings indicate that SP with MnSO_4_ and Mn_2_O_3_-NPs stimulates the processes that occur prior to germination and thus reduces the time for radicle emergence. In both applications (i.e., SP and +F), none of the Mn treatments did show adverse phytotoxic on *A. annua* growth at morpho-physio and biochemical levels except for Mn_2_O_3,_ which delayed germination and further plant growth, subsequently. Besides, from physio-biochemical data, it can be inferred that the general mechanism mode of action of Mn is mainly attributed to induce the photosynthetic processes, stimulate the superoxide dismutase (SOD) activity, and up-regulation of proline and phenolic compounds. Therefore, our results showed that both enzymatic and non-enzymatic antioxidants could be influenced by the application of Mn treatments in a type-dependent manner. In general, this study revealed that Mn_2_O_3_-NPs at the tested condition could be used as biostimulants to improve germination, seedling development and further plant growth. However, they are not as effective as MnSO_4_ treatments. Nonetheless, these findings can be used to consider and develop Mn_2_O_3_-NPs priming in future studies to improve seed germination and seedling quality in plants

## 1 Introduction

Nanotechnology is currently starting to govern various aspects of human life and getting to shape the near future of a number of disciplines, such as physics, biology, material science and so on. For the time being, some studies have displayed an optimistic side of this technology in plant science as well. Nanoparticles (NPs) have been explored as “multifunctional particles” carrying agrochemicals such as herbicides, fertilizers, and have been recently delivered genes, targeting the specific cell organelles (Das et al., 2009; Ahmar et al., 2021). Plants can benefit through the appropriate usage of nanomaterials at a lower amount as they have shown higher efficiency and probably lower contamination (Pérez-de-Luque, 2017). Although this scenario is something scientific society is willing to believe in, but it needs extra and persuadable work to prove it. Being used in the right place and at the right time with an effective method, nanoparticles can improve plant performance, particularly at the early stages of development. The expected effects have been reported to be increased germination, healthier seedlings and higher biomass in terms of growth indices (Szőllősi et al., 2020; Wu and Li, 2021). The NPs-induced alteration is highly influenced by many factors, such as the nature of NPs themselves, dosages, plant species and the application procedure (Khan et al., 2019; Salehi et al., 2021a; Salehi et al., 2021b). In this regard, studies are trying to provide evidence on how NPs work, but getting the whole overview is extremely complicated. Part of this complexity in plants is dealing with thousands of different plant species. Another reason is that most of the studies have been performed in some plant models or agricultural-related crops, and also the experiments have been limited to the early seedling stages (Pérez-de-Luque, 2017).

The application of oxide metal ions and metal NPs applied at lower dosages has been found to be eco-friendly, reduce oxidative stress, positively affect germination index, and promote biosynthesis of photosynthetic pigments, various enzymatic and non-enzymatic molecules, which is reflected in improved plant growth and development (Salehi et al., 2019; Kumari et al., 2022). Some studies have reported similar effects of NPs to their ionic and bulk counterparts (Jeevanandam et al., 2018; Pullagurala et al., 2018a; Pullagurala et al., 2018b); however, contrary results have been obtained by some others, indicating different effects of NPs due to their specific characteristics (Santiago et al., 2020; Kralova and Jampilek, 2021). On the other hand, it is not fully accepted that NPs forms have much more influence on plants. To achieve higher yield by NPs, it is vital to explore which kind of NPs and at which concentrations boost plant growth for individual plant species. Crop plants have been widely investigated to assay NPs effects from both negative and positive perspectives. However, how NPs affect medicinal plants is another key aspect of plant-NPs interactions, because it will affect the practical application of NPs.

Medicinal plants, considered as rich resources of ingredients either in pharmaceutical and food science, play not only an important role in medicine but also as phytochemical building blocks for the development of new drugs (Zahra et al., 2020). These valuable plants should generally not be cultivated in contaminated medium, and chemicals applied to promote their growth should be kept to a minimum in order to avoid showing toxic signs. Moreover, it is critical to introduce safe and favorable conditions for their germination and development. Regarding this, using NPs has recently been interested due to using a lower amount of active ingredient to obtain the biological results as similar to their bulk and ionic counterparts. To the best of the authors’ knowledge, only a small number of researches on medicinal plants have been incorporating NPs. So far, several studies have shown the beneficial effects of NPs such as Ag, Cu, Fe, Zn and Ti on germination, seedling performance, and biochemical compounds in a limited number of medicinal plants like *Salvia officinalis* (Moazzami Farida et al., 2020), *Rosmarinus officinalis* (Hadi Soltanabad et al., 2020), *Mentha longifolia* (Talankova-Sereda et al., 2016), *Origanum vulgare* (Du et al., 2018), *Cuminum cyminum* (Sabet and Mortazaeinezhad, 2018), *Dracocephalum kotschyi* (Nourozi et al., 2019), *Tanacetum parthenium* (Shahhoseini et al., 2020), *Stevia rebaudiana* (Velázquez-Gamboa et al., 2021) and *Mentha piperita* (Ahmad et al., 2018). It has been reported that mild oxidative stress induced by NPs at the level of nontoxic concentrations can stimulate seed germination and enhance plant performance to achieve higher yields (Landa, 2021). In the case of medicinal plants, this event may boost the biosynthesis of valuable secondary compounds as well.

Manganese (Mn) is an essential micro-metalloid sustaining metabolic roles within cell compartments, which is led to plant growth and development. The metal has various roles in plant’s metabolic processes, including being an important metalloenzyme cofactor in photosynthetic machinery, respiration, detoxification of reactive oxygen species (ROS), and hormone transduction signaling (Alejandro et al., 2020). Nanoscale Mn compared to conventional ionic and bulk Mn species found to be a better source of Mn and less phytotoxic, and thus more effective in minimizing internal and external stresses in plants (Ye et al., 2019). A recent study reported that watermelon seeds primed with MnO-NPs show less phytotoxicity compared to the bulk forms (KMnO_4_ and Mn_2_O_3_). The findings indicated that MnO-NPs at 20 mg L^-1^ significantly affect the chlorophyll and antioxidant metabolites, while at ≤ 40 mg L^-1^, phenolic and phytohormone profiles were altered (Kasote et al., 2021). Although there is evidence of promoting and alleviating the effect of different traditional types of Mn on plant growth, the unknown effects related to Mn_2_O_3_-NPs medicinal plant interaction need to be discovered.

*Artemisia annua*, an herbal plant, is the only commercially available resource for artemisinin biosynthesis, which has been applied to cure malaria for so long. Besides, the extracted compounds from *A. annua* have also been reported to have promising therapeutic effects for diabetes, tuberculosis, and, recently corona virus (Shen et al., 2022). These findings further accelerate the global demand for huge amounts of *A. annua* plants and, therefore, their functional compounds. Taking this into account, the use of nutripriming could be an effective strategy to improve the growth and development of A. annua, thus providing higher yields. Since, soil application of Mn is often not effective due to its conversion to plant-unavailable Mn oxidase, in this study, we have used seed priming and foliar application of Mn_2_O_3_-NPs and their counterparts to assay and comparison their mode-of-action. Seed priming has been proven to be an effective method to stimulate seed germination rate and seedling emergence, leading to high-quality seedlings and improved plant growth (Adhikari et al., 2022). Nutri-seed-priming enhances water uptake into seeds, triggering starch metabolism and, therefore, faster seed germination (Nile et al., 2022). On the other hand, foliar application with NPs-based nutrients provides a faster and more efficient way to trap essential nutrients (Salehi et al., 2018; Salehi et al., 2021a). Based on these reports, we hypothesis that a combination of seed priming and foliar application has a better effect on plant growth.

## 2 Material and methods

### 2.1 Mn_2_O_3_ NPs preparation and characterization

Particulate Mn_2_O_3_-NPs with a size of 30 nm, 99.2% purity, surface area of 150 m^2^ g^-1^, and true density of ~0.35 g^-1^ cm^3^ were obtained from NANOSANY Co. Ltd, Mashhad, Iran. Their physico-chemical properties, including scanning electron microscopy (SEM), transmission electron microscopy (TEM) and powder X-ray diffraction (XRD) have been presented in **Figure S1**. MnSO_4_, MnCl_2_ and Mn_2_O_3_ were also purchased from Sigma Aldrich (Germany).

### 2.2 Experimental design, phyto-safety of Mn_2_O_3_-NPs assay, and sample harvesting

**Figure 1:**
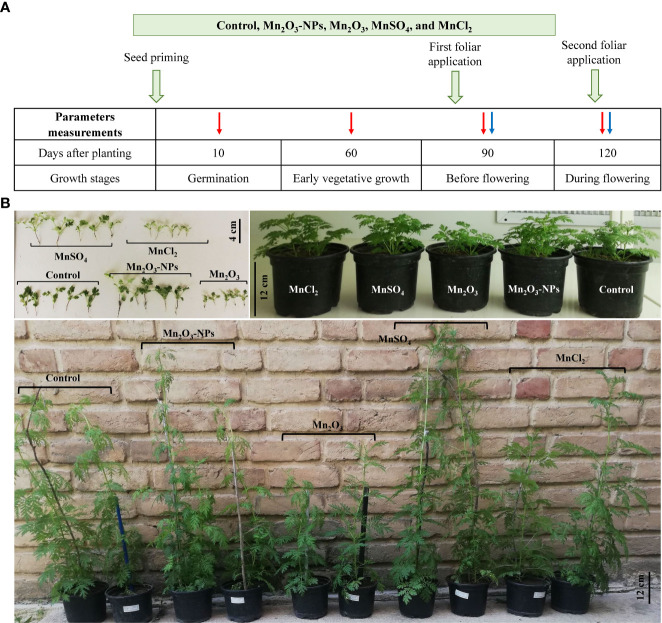
Schematic design of the treatments in the experiment **(A)**; the orange arrows indicate the time of treatments; the red and blue arrows show the parameters measured time-intervals in SP and SP+F conditions, respectively, to investigate the effects of Mn treatments in *A. annua*. Figure **(B)** is related to the plant growth at seedlings (top-left), 60 days (top-right) and 120 days (bottom) after planting. In the bottom figure, the post labeled in white are related to the SP condition.

*Artemisia Annua* seeds were obtained from Plant Bank, Iranian Biological Resource Center (IBRC, Tehran, Iran). This species has been recommended for high yields of bioactive compounds and, therefore, is applicable in clinical industry (Dogan et al., 2022). The experiment presented here was carried out using a completely randomized design in 2022 at Laboratory of Plant Cell Biology, Department of Biology, Bu-Ali Sina University, Hamedan, Iran. The Mn_2_O_3_-NPs concentration and time used in this experiment were determined after a phyto-safety germination test. Briefly, the sterilized seeds were primed with three different concentrations (25, 50, and 100 mg L^-1^) of Mn_2_O_3_-NPs for 3 and 6 h and incubated in a germinator chamber for 10 days. The concentrations were selected according to available studies that highlight these levels cannot be considered phytotoxic in plants (Tian et al., 2018; Li et al., 2020; Kasote et al., 2021). The results of germination test showed that seed priming with 50 mg L^-1^ for 3 h causes a higher germination percentage (**Figure S2**). In this experiment, we used two applications (seed priming (SP) and a combination of seed priming+foliar spray (SP+F). The latter was done to evaluate the possible further improve plant growth under Mn treatments. Following germination, harvesting for analyses was carried out at several developmental stages including early vegetative (60 day), before flowering (90 day) and during flowering (120 day) stages. The schematic design of the experiment is shown in **Figure 1A**.

After selecting two factors (concentration and seed priming (SP) time), to assay seed germination, Mn_2_O_3_-NPs, MnSO_4_, MnCl_2_ and Mn_2_O_3_ suspensions were individually prepared in Milli-Q water and sonicated for 30 min before use to avoid aggregation. Ionic and bulk compounds were used as a positive control to assay the effect of different species of Mn on plant growth. *A. annua* seeds were presoaked in 2 mL of each priming solution for 3 h in a shaker to prime all seeds equally. Seeds soaked in Milli-Q water were used as hydro-primed control. After drying, primed seeds were divided into several groups and sown in a plug tray filled with coco perlite-vermiculite for germination and early seedling development. The germination-related parameters were measured after the whole germination for 10 days after sowing. After 30 days, the healthy seedlings from each treatment were transformed into growing pots (13 cm diameter and 15 cm height) containing 2 kg of local soil (pH=7.66, electrical conductivity =0.21ds/m, cation exchange capacity =28.17 meq/100g, CaCO_3_ = 24.45%, organic matter=3.78%, and organic carbon=2.19%). Pots were then incubated in the environmental growth chamber (24 ± 5 temperature, 70% relative humidity and 12h photoperiod) for 120 days, till the flowering stage. These pots were already divided into two main groups (one for SP and other for SP+F). At least 50 seeds were used per group. The depth of the holes was 4 cm. The first group of plants was used to investigate the effect of Mn_2_O_3_-NPs SP method and then compared with other counterparts. The two-month-old plants grown in SP condition were selected for spray application (second group). Plants were sprayed with 5 mL suspensions at 75 and 105 days after planting (10 mL in total). During the foliar spray, an aluminum wrap was used to cover the soil surface to avoid contamination. Overall, samples were harvested three times at 60, 90 and 120 days after transplanting, which are determined as early vegetative seedlings, before and during flowering development stages. Samples were thoroughly washed with milli-Q water to remove Mn residues prior to freezing at -80°C for further analysis. It is worth mentioning that plants were regularly watered to keep almost 60% of soil field capacity. In this experiment, no additional fertilizer was added. Five replicates (pots) were used for each treatment.

### 2.3 Germination and growth parameters analysis

Germination test was conducted in the dark at 25°C for 10 days. Sets of 20 primed seeds from each treatment were placed in Petri dishes and incubated in the germination chamber (25 ± 2°C, 80% humidity). Germination rates were assessed by counting the number of germinated seeds (1 mm radicle emergence) at every 24 h intervals. The germination and initial seedling growth-related variables including germination index (GI), germination percentage (GP), relative seed germination (RSG), and coefficient of the velocity of germination (CVG) were measured according to the previously reported formulas (Sobarzo-Bernal et al., 2021). At each developmental stage, plant height was measured per pot from the ground level to the panicle tip. In addition, dry weight was also measured at the end of the trial.

### 2.4 Determination of leaf relative water content and electrolyte leakage

Leaf relative water content (RWC), reflecting the balance between water supply and transpiration rate, was used to assay water status in *A. annua* plants after Mn treatments. In detail, two fully expanded and healthy leaves of three plants per replicate were cut, weighted and then floated in 20 mL distilled water for 24 h at room temperature for full hydration. The leaves were then blotted on a dry surface with filter paper. Following that, turgid and dry weights (at 70°C for 2 d) were measured. RWC was calculated following the equation: RWC = (FW-DW)/(TW-DW)×100. Where FW, DW, and TW are fresh weight, dry weight, and turgor weight, respectively.

Electrolyte leakage (EL), as cell membrane injury, was measured following the method of Lutts, 2004 (Lutts et al., 2004). Briefly, three fresh, mature and healthy leaves (the 4^th^ leaf from the tip) per plant were chosen, washed with distilled water until removing surface contamination and then cut into small pieces (1 cm). The leaf segments were placed in individual test tubes containing 15 mL of distilled water. After 24 h incubation at 25°C (room temperature), the electrical conductivity of the solution was read (EC_1_). EC_2_ was read after autoclaving (121°C for 15 min) and cooling down the solution to room temperature. The EL was calculated as the following equation: EL = (EC_1_/EC_2_)×100.

### 2.5 Determination of lipid peroxidation markers (MDA and H_2_O_2_content)

Hydrogen peroxidase (H_2_O_2_) and malondialdehyde (MDA) concentrations are the widely used method to analyze lipid peroxidation in plants. Potassium iodide (KI) was used to assay hydrogen peroxide (H_2_O_2_) level (Alexieva et al., 2001). Briefly, leaf tissues (0.5 g) were grinded in mortar and pestle with liquid nitrogen and thoroughly homogenized with 5 mL 0.1% (w/v) trichloroacetic acid (TCA). The homogenate mixture was centrifuged at 12000 revolutions per minute (rpm) at 4^°^C for 15 min. Reaction mixture consisted 0.5 mL extract supernatant, 0.5 mL 10 mM potassium phosphate buffer (pH 7.0) and 1 mL reagent (1 M KI w/v in fresh double-distilled water). The absorbance of reaction was measured at 390 nm and the content of H_2_O_2_ was calculated using a standard curve prepared with known concentrations of H_2_O_2_. The blank consisted of 0.1% TCA without leaf extract.

The same extract used for H_2_O_2_ measurement was also used for estimating the MDA level. To initiate the reaction, 225 µL of the extract was incubated with 1.5 mL of 20% TCA containing 0.5% thiobarbituric acid (TBA) for 40 min at 95 ^°^C. The reaction was stopped by cooling it quickly in an ice bath, then centrifuged for 10 min at 6000 rpm to clear the reaction mixture. The absorbance of the supernatant was read at 532 nm and corrected for unspecific turbidity by subtracting the value from the absorbance at 600 nm. The MDA concentration was calculated using an extinction coefficient of 155 mM^-1^ cm^-1^ and expressed as mmol mg^-1^ fresh weight (Stewart and Bewley, 1980).

### 2.6 Chlorophylls and total carotenoid content

The spectral (Biowave II, England) determination of chlorophyll (Chl) a, b, and total, as well as total carotenoids of *A. annua* leaves (500 mg) was performed using 80% acetone (10 mL) according to the method of Arnon (Arnon, 1949). The related absorbance was taken at 663, 645 and 470 nm, respectively. The content of Chl a = 12.25 × A_664_–2.55 × A_645_, Chl b = 20.31 × A_645_–6.91 × A_664_, total Chl = 17.76 × A_645_ +7.37 × A_664_, and carotenoids=A_470_+(0.114×A_663_) -(0.638×A_645_) were then calculated and data were reported as mg pigments per g fresh weight.

### 2.7 Total phenolic and flavonoid content

To assay the total phenolic and total flavonoid content, the methanolic extract was prepared by grinding and homogenizing 300 mg of fresh tissues with 3 ml methanol 80% (0.1% formic acid). Then the mixture was centrifuged at 6000 rpm for 10 min at room temperature. The supernatant was used for further analyses.

The total phenolic content (TPC) was estimated by Folin-Ciocalteu (F-C) method (Chun et al., 2003) with a little modification. Briefly, 100 µL of the metabolic extract was mixed with 400 µL water and 500 µL F-C reagents (10 fold diluted with distilled water). The mixture was stand at room temperature for 5 min, and afterward, 1 mL of sodium carbonate (7.5%) was added to the mixture and then incubated in darkness for 2h. The absorbance of mixture was recorded at 765 nm. The TPC concentration was expressed in terms of gallic acid equivalent (mg gallic acid/g of fresh mass).

An aluminium chloride (AlCl_3_) colorimetric assay was applied to measure the total flavonoid content (Zhishen et al., 1999). The mixture contained 500 µL methanolic extract, 2 mL distilled water, and 150 µL 5% sodium nitrate was left at room temperature for 10 min. Thereafter, 300 µL 10% AlCl_3_ was added and incubated for another 15 min. The absorbance was read at 510 nm versus a blank. Total flavonoid content was expressed as mg catechin (CA) equivalents per g of fresh weight.

### 2.8 Proline content

The proline content was assayed to monitor the physiological status of *A. annua* with a standard ninhydrin-based method using a cuvette spectrophotometer (Bates et al., 1973). Briefly, fresh tissues (0.2 g) were homogenized in 5 mL of 3% aqueous sulfosalicylic acid and left for 1h to complete the extraction. The solution was centrifuged at 6000 rpm for 10 min. The mixture containing 2 mL of supernatant, 2 mL glacial acetic acid and 2 mL acidic ninhydrin was then boiled in a water bath for 60 min. The reaction was stopped by placing the mixture in an ice bath and afterward 4 mL of toluene was added and mixed vigorously using a vortex. After reaching room temperature the absorbance was read at 520 nm against toluene, as blank. The proline content was calculated using a standard curve from 20-100 µg/mL of L-proline.

### 2.9 Soluble protein content and enzyme assay

Crude enzyme extracts were prepared by grinding 500 mg of leaves tissue using liquid nitrogen and homogenized thoroughly in 5 mL of potassium phosphate buffer (PPB) (100 mM, pH 7.8) containing 1 mM EDTA and 1% w/v polyvinylpyrrolidone (PVP). The homogenate was then centrifuged at 10000 rotations per min (rpm) at 4 °C for 15 min to remove debris. The supernatant was used for further analysis. Bradford dye-binding method was used to assay soluble proteins.

Catalase (CAT, EC 1.11.1.6) activity was assayed by estimating the initial rate of disappearance of H_2_O_2_ followed by Beers and Sizer method (Beers and Sizer, 1952). The reaction mixture contains 2.8 mL of 50 mM PPB, 0.1 mL of enzyme extract, and 30 µL of 15 mM H_2_O_2_. CAT activity was measured in 1 minute at 240 nm using a UV-visible spectrophotometer. One enzyme unit corresponds to the amount of enzyme required to break down one µM of H_2_O_2_ min^-1^ or mg^-1^ protein (extinction coefficient of 34 mM cm ^-1^).

Polyphenol oxidase (PPO, EC 1.14.18.1) activity was assayed by the method of Raymond et al. (Raymond et al., 1993). The enzyme was assayed by putting 2.5 mL of assay buffer (PBP 50 mM, pH 7) and 0.2 ml of pyrogallol (20 mM) in a cuvette of 5ml capacity. The assay reaction was initiated by adding 0.1 mL of enzyme extract, followed by recording the change in absorbance at 420 nm wavelength, simultaneously for 3 min. The enzyme activity was expressed as pyrogallol oxidized after 3 min per mg protein (unit mg^-1^ protein).

To assay ascorbate peroxidase (APX, EC 1.11.1.11) activity, a total reaction mixture containing 2.5 mL PPB (50 mM, pH 7), 30 µL H_2_O_2_ (0.1 mM), 300 µL ascorbic acid (0,5 mM), 30 µL EDTA (0.1 mM), and 150 µL enzyme extract was put into a cuvette. The decrease in absorbance was recorded at 290 nm for two min. Extinction coefficient of 2.8 mM cm^-1^ was used to calculate the amount of ascorbate oxidized (unit mg^-1^ protein) (Jebara et al., 2005).

Superoxide dismutase (SOD, EC 1.15.1.1) activity was measured by screening enzyme ability to inhibit the photochemical reduction of nitrotetrazolium blue (NBT), according to Fu and Huang protocol (2001). Briefly, each 3 mL of reaction mixture contained 50 µl of enzyme extract, 1 mL NBT (63 μM), 1 mL riboflavin 1.3 μM, 750 µL methionine (13 mM), and 250 μL EDTA (0.1 mM). Reaction was initiated by exposing the test tubes under fluorescent lamp for 10 min and stopped by switching off the lamp, anf then the absorbance was read at 560 nm. The blank reaction mixture was kept in the dark the whole time. SOD activity was expressed in units per min per mg protein. One unit of SOD was defined as the amount of enzyme that inhibits 50% of NBT photoreduction.

### 2.10 Statistical analysis

All estimated data were analyzed using the SPSS program (SPSS, Chicago, IL, USA). A one-way analysis of variance (ANOVA) and Duncan comparison test were performed to compare the mean values of the control plants verse the Mn-treated plants. Data are represented as mean ± standard error, and significant differences were determined at P ≤ 0.05. A multivariate analysis, including a principal component analysis (PCA) and Pearson’s moment-product correlations were further performed with individual values of all the parameters. A heatmap and cluster analysis was performed using ClustVis (www.biit.cs.ut.ee/clustvis/).

## 3 Results

### 3.1 The effect of different Mn forms on germination and plant growth

**Figure 2:**
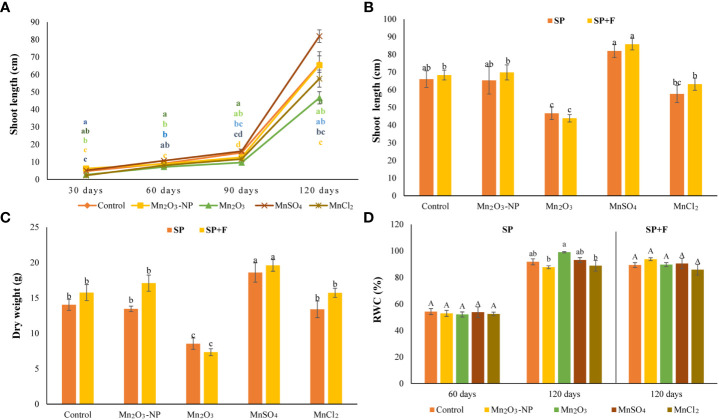
Shoot length of plants primed with different Mn forms (grown in SP condition) at different time intervals **(A)**; the comparison of shoot length **(B)** and dry weight **(C)** of plants grown in SP and SP+F condition at 120 days; relative water content **(D)**. Data points and error bars represent mean and S.E. (n = 3). Values with different letters indicate significant differences at the P < 0.05 level between different Mn forms.

The results showed germination-related parameters, including CVG, RSG, GI, and GP showed significant differences between treatments (**Table 1**). Seeds treated with MnSO_4_ and Mn_2_O_3_-NPs exhibited a mean value of GI and RSG parameters higher than 100%. In addition, the germination percentage in these two treatments were significantly higher than in others. These results represent a biostimulant effect of Mn_2_O_3_-NPs and particularly MnSO_4_ on germination. However, seeds treated with MnCl_2_ and Mn_2_O_3_ showed decreased mean values in all parameters. Notably, Mn_2_O_3_, GI, RSG, and CVG parameters were decreased by 77%, 51%, and 31% compared to the control, respectively (**Table 1**). Additionally, Mn_2_O_3_ delayed the onset of germination by about 6-8 days. **Figure 2** exhibits the shoot length and dry weight of *A. annua* plants after priming and foliar spraying of different Mn forms. At almost every developmental stage, shoot length was improved by MnSO_4_ treatment compared with control and other treatments (**Figures 1**, **2**). However, Mn_2_O_3_-NPs only had the inducing effect on shoot length at 30 days after planting (an increase of 24% compared to control), and after that, it was the same as the control condition.

**Table 1:** Germination-related parameters of *A.annua* seeds primed with different Mn species.

Treatments	Germination Index	Relative Seed Germination (%)	Coefficient of Velocity of Germination	Germination Percentage (%)
**Control**	100.00 ± 00b	100.00 ± 0.00a	43.50 ± 4.41ab	81.11 ± 2.94a
**Mn_2_O_3_-NPs**	115.62 ± 7.03b	107.41 ± 7.41a	46.06 ± 2.49a	84.44 ± 1.11a
**Mn_2_O_3_**	21.31 ± 3.13c	48.48 ± 9.74b	29.93 ± 3.53c	32.22 ± 2.94c
**MnSO_4_**	157.70 ± 23.12a	123.91 ± 12.94a	52.41 ± 2.88a	88.89 ± 2.94a
**MnCl_2_**	51.27 ± 4.25c	69.61 ± 5.83b	34.71 ± 2.71bc	63.33 ± 3.85b
**Sig**	**	**	**	**

**means significant at p ≤ 0.005.

Means ± SDs with different letters in each column indicate significant statistical differences (P≤, 0.05).

Under Mn_2_O_3_ treatment, a significant decrease (42%) in plant growth was noted, as can be explained by lower and late germination. The comparison of plant growth after 120 days after planting in two application ways (i.e., SP and SP+F) showed the same trend, as MnSO_4_ and Mn_2_O_3_ caused an increased and decreased, respectively. Analysis of variance showed that there is no significant difference between the two applications but treatments had significant effects (P<0.005) on plant growth. The RWC didnot show a remarkable difference between treatments and also applications (**Figure 2D**).

### 3.2 Changes in electrolyte leakage and cell injury indicators

**Figure 3:**
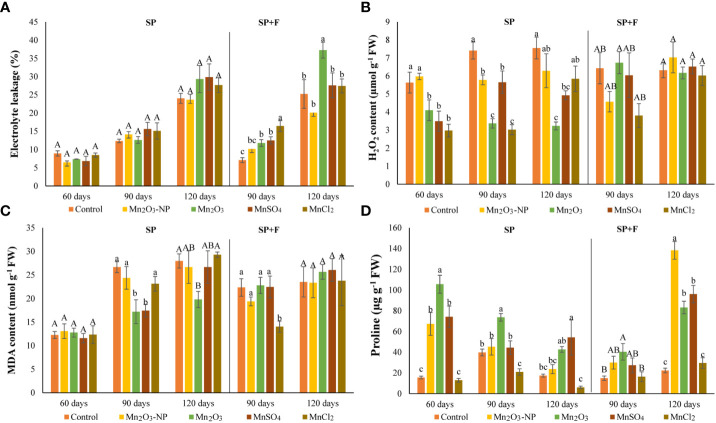
Electrolyte leakage **(A)**, H_2_O_2_ **(B)**, MDA **(C)**, and proline **(D)** contents in *A. annua* primed with different Mn forms at different durations. Data are mean ± SE (n=3). The different letters indicate statistically significant differences between Mn treatments within the same group at p<0.05. Capital letters represent no significant differences, statistically.

The plants treated with Mn species were checked for electrolyte leakage (EL). The results showed that none of Mn treatments significantly affected EL in plants grown under SP conditions (**Figure 3A**). However, EL increased during plant growth, particularly at 120 days which could be due to the intrinsic changes of plant itself, like the senescence process. In SP+F application, at 90 days when foliar spray was done, EL increased slightly in Mn treatments compared to control. These changes at 120 days also showed significant differences between all Mn forms in which Mn_2_O_3_ induced higher EL, while NPs minimized it. Generally, a significant application-dependent change was observed in which EL was lower in SP+F compared to SP, indicating a mild alleviating effect of Mn foliar spray.

Furthermore, the MDA and H_2_O_2_ contents, known as cell injury indicators, were also monitored (**Figures 3B, C**). The content of MDA and H_2_O_2_ in almost all treatments in both applications was increased as plant grows, which originated from the internal metabolism. However, their content in Mn_2_O_3_ treatment in plants grown under SP conditions was lower compared to control and other treatments. This was expected because of the late germination and, thus, the stunned vegetative growth. Contrarily, a decreased MDA and H_2_O_2_ in some treatments like MnSO_4_ and Mn_2_O_3_-NPs compared to control can be attributed to the positive effect of these Mn forms. Regarding SP+F application, the differences between treatments were not statistically significant. However, the first spray of Mn_2_O_3_-NPs and MnCl_2_ treatments showed slightly decreased MDA and H_2_O_2_ content. The multivariate analysis showed no significant differences in MDA and H_2_O_2_ changes in two applications except for H_2_O_2_ at 120 days.

The increased proline accumulation at 60 days of plants grown in SP condition was observed mainly in Mn_2_O_3_ treatment (+85%) (**Figure 3D**). It has also shown a significant increase with NPs and MnSO_4_ treatments compared to control. However, a declining trend was observed over time at 90 and 120 days plants. Although the proline content was increased in all Mn forms (except MnCl_2_) at 120 days, its level was lower than 60 days’ treatments. Lower proline accumulation at 120 days after priming may be attributed to physiological adaptation. Moreover, a general decrease and increase were observed at 90 and 120 days’ plants after foliar application of Mn forms, respectively. In detail, proline content was increased in Mn_2_O_3_-NPs (+83%) and MnSO_4_ (+76%) treatments compared with control plants (**Figure 3D**). Accordingly, analysis of variance showed a significant application-dependent response of proline content in plants cultivated under SP and SP+F conditions.

### 3.3 Protein content and Antioxidant enzymes response

**Figure 4:**
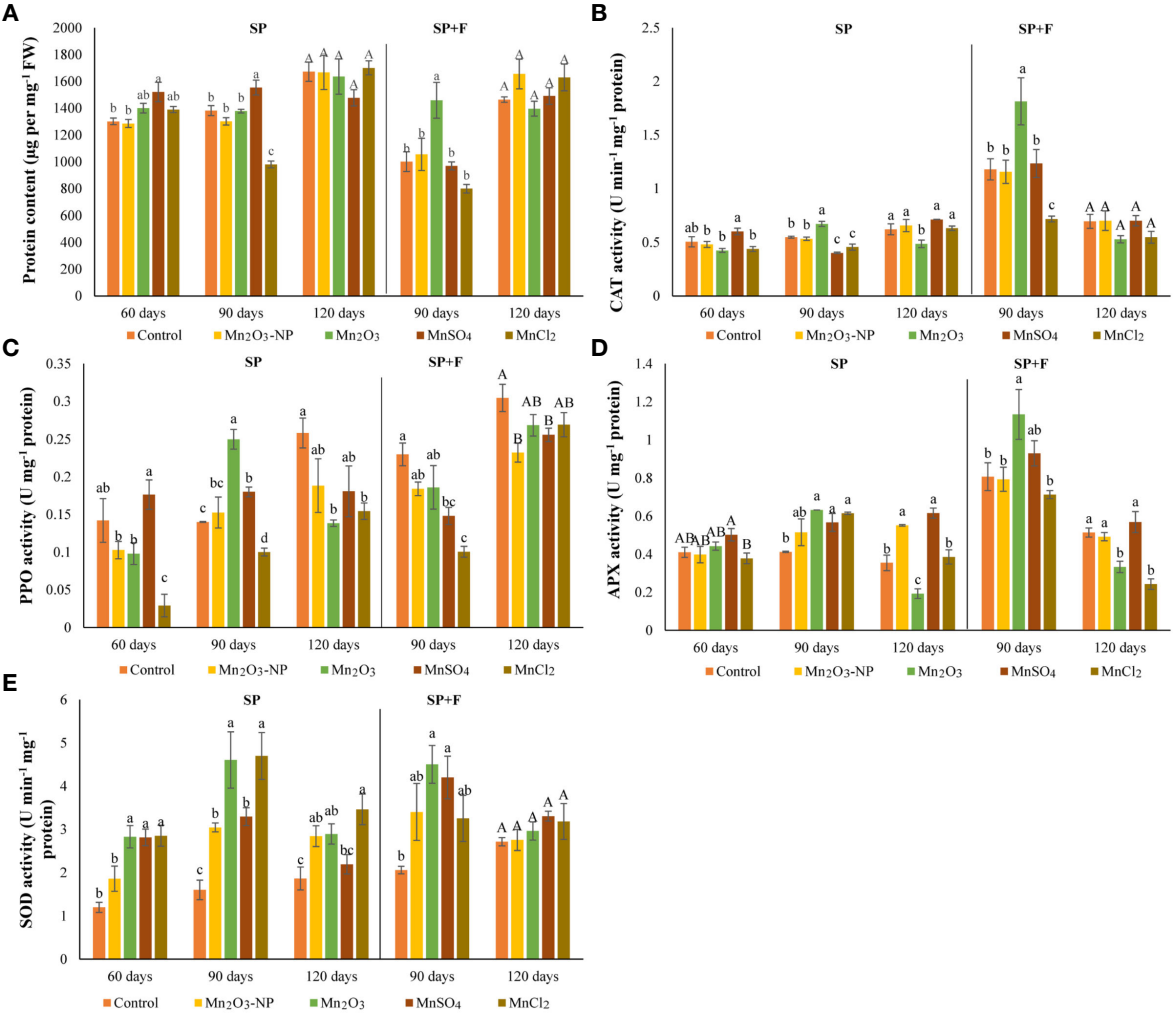
Protein content **(A)** and antioxidant enzymes (PPO **(B)**, APX **(C)**, SOD **(D)**, and CAT **(E)**) of *A. annua* under different Mn treatments using SP and a combination of SP+F at different time intervals.

The protein content has also differed at tested time intervals, but the highest content was observed at 120 days’ plants. In general, foliar application of Mn treatments at 90 days decreased protein content compared to the relevant treatments of SP only (**Figure 4A**). The oxidative status of the plants treated by different Mn forms grown in two conditions (SP and SP+F) was surveyed by measurement of CAT, PPO, APX, and SOD activities (**Figures 4B-D**). Each enzyme had a specific trend of changes based on the Mn form, application type and plant growth phase. For example, CAT activity did not show significant differences between all three developmental stages of plant growth in SP conditions over time. However, there was an Mn-dependent response as MnSO_4_ and Mn_2_O_3_ treatments increased the activity of CAT at 60 and 90 days, respectively. Regarding SP+F application, spraying of Mn treatments (except for MnCl_2_) and even water in control increased CAT activity at 90 days compared with their relevant treatments in SP (an average increase of 60%) (**Figure 4B**). Accordingly, the highest level of CAT activity was observed in Mn_2_O_3_ treatment (a 34% increase compared to the control).

PPO activity showed a general decrease in Mn treatments (except for MnSO_4_) compared to control in 60 days’ of plants grown in SP condition. However, an overall increase was observed over time as its activity was manifested by % 45 in 90 days’ of plants treated with Mn_2_O_3_ (**Figure 4C**). At the same time, foliar application of NPs increased the PPO activity by 34% compared to its relevant treatment in plants grown only in SP conditions. The same trend of changes was also found for ionic forms of Mn but not Mn_2_O_3_. At 120 days’ plants, all Mn forms increased PPO activity, but an overall decline was observed compared to 90 days’ plants. A similar trend of increase was also observed for APX activity at 90 days’ of plants grown under both SP and SP+F applications (**Figure 4D**). The highest level of APX activity was attributed to Mn_2_O_3_ treatment (a 44% increase compared with its relevant at 90 days in SP condition). At 120 days’ plants, APX activity was decreased compared to 90 days’ plants. Additionally, a general decline was observed for Mn_2_O_3_ and MnCl_2_ treatments. An interesting trend of changes was observed for SOD activity, as its activity was upregulated in all Mn treatments (**Figure 4E**). In general, the results showed that SOD activity was significantly enhanced by Mn_2_O_3_ and MnCl_2_ treatments. There was no remarkable alteration of SOD activity between SP and SP+F applications.

### 3.4 Photosynthetic pigments, total phenolic and flavonoid content

The Chl indices (Chla, b, total, a/b ratio, carotenoids, and Chlt/carotenoid ratio) have been represented in **Table 1**. A noticeable increase was observed in photosynthetic pigments, including Chl a, Chl b, total Chl, and carotenoids in both applications, especially at 90 days after planting. But their content was decreased in 120 days’ plants compared to 90 days’ plants which may be due to the senescence process in the plant. All Mn forms, particularly Mn_2_O_3_-NPs and Mn_2_O_3,_ showed a positive effect on pigment synthesis. The multivariate analysis also showed a significant difference between applications, indicating a distinctive effect of seed priming and foliar spray on photosynthetic pigments. However, a general increase in Mn treatments was noticed at all developmental stages, even 120 days, highlighting the positive effect of Mn on pigments synthesis. Regarding the ratio of Chl a to Chl b, higher values were recorded for plants treated with Mn forms compared to control in SP condition, whereas the opposite trend was observed for SP+F condition. A general increase of Chl t/carotenoid ratio was mainly found in Mn treatments compared to control at 60 and 90 days’ plants in both applications (**Table 2**). In total, it was found that all different Mn forms, regardless of the type, play an important role in improving photosynthetic pigments.

**Table 2:** The effect of different forms of Mn on photosynthetic pigments, total phenol and total flavonoid content in *A. annua* plants at three different times using two applications (SP and SP+F).

	Chl a (mg g^-1^ FW)	Chl b (mg g^-1^ FW)	Chl t (mg g^-1^ FW)	Carotenoids (mg g^-1^ FW)	Chl a/Chl b ratio	Chl t/Car ratio	Total phenol (µg GAE g^-1^ FW)	Total flavonoids (mg CA g^-1^ FW)
SP								
60 day								
C	0.93 ± 0.18c	0.76 ± 0.09b	1.69 ± 0.27c	1.29 ± 0.05A	1.21 ± 0.11b	1.31 ± 0.22c	67.7 ± 8.8a	1.41 ± 0.03c
Mn_2_O_3_-NPs	1.01 ± 0.08c	0.77 ± 0.04b	1.78 ± 0.12c	1.32 ± 0.02A	1.31 ± 0.04b	1.34 ± 0.08c	36.0 ± 0.6b	1.37 ± 0.08a
Mn_2_O_3_	1.59 ± 0.10ab	1.04 ± 0.05ab	2.63 ± 0.15ab	1.46 ± 0.04A	1.53 ± 0.05a	1.80 ± 0.11ab	29.3 ± 4.4b	1.13 ± 0.07c
MnSO_4_	1.33 ± 0.04bc	0.94 ± 0.02b	2.27 ± 0.06bc	1.49 ± 0.05A	1.42 ± 0.02ab	1.53 ± 0.08bc	61.7 ± 3.9a	1.79 ± 0.04ab
MnCl_2_	1.90 ± 0.20a	1.35 ± 0.20a	3.26 ± 0. 4a	1.52 ± 0.14A	1.42 ± 0.06ab	2.13 ± 0.09a	30.7 ± 1.2b	1.38 ± 0.06bc
90 day								
C	1.71 ± 0.29b	1.28 ± 0.23b	2.99 ± 0.53b	1.68 ± 0.08B	1.34 ± 0.02A	1.75 ± 0.23A	106.0 ± 0.6b	2.34 ± 0.06b
Mn_2_O_3_-NPs	2.35 ± 0.04a	1.83 ± 0.05a	4.19 ± 0.09a	1.89 ± 0.01AB	1.28 ± 0.02A	2.21 ± 0.04A	78.1 ± 3.1c	1.95 ± 0.07c
Mn_2_O_3_	2.46 ± 0.02a	1.87 ± 0.10a	4.33 ± 0.08a	2.15 ± 0.21A	1.32 ± 0.09A	2.06 ± 0.24A	77.3 ± 3.2c	2.54 ± 0.04a
MnSO_4_	2.20 ± 0.02a	1.57 ± 0.03ab	3.77 ± 0.05ab	1.76 ± 0.04B	1.41 ± 0.02A	2.15 ± 0.02A	157.9 ± 8.1a	2.31 ± 0.04b
MnCl_2_	2.18 ± 0.02a	1.56 ± 0.04ab	3.76 ± 0.07ab	1.69 ± 0.03B	1.40 ± 0.02A	2.21 ± 0.01A	83.6 ± 7.4c	2.01 ± 0.06c
120 day								
C	1.59 ± 0.05A	1.08 ± 0.01A	2.67 ± 0.05A	1.76 ± 0.02A	1.46 ± 0.03c	1.52 ± 0.04B	199.5 ± 31.5b	1.92 ± 0.01B
Mn_2_O_3_-NPs	1.74 ± 0.10A	1.05 ± 0.05A	2.79 ± 0.16A	1.65 ± 0.02A	1.67 ± 0.01a	1.69 ± 0.07AB	276.0 ± 8.7a	1.87 ± 0.01B
Mn_2_O_3_	1.65 ± 0.11A	1.04 ± 0.08A	2.69 ± 0.20A	1.42 ± 0.29A	1.58 ± 0.02b	2.02 ± 0.30A	188.0 ± 38.7b	1.96 ± 0.02B
MnSO_4_	1.70 ± 0.12A	1.09 ± 0.06A	2.79 ± 0.18A	1.73 ± 0.02A	1.56 ± 0.02b	1.61 ± 0.08AB	328.5 ± 2.6a	1.99 ± 0.03AB
MnCl_2_	1.73 ± 0.11A	1.18 ± 0.07A	2.91 ± 0.18A	1.85 ± 0.07A	1.47 ± 0.01c	1.57 ± 0.04AB	312.0 ± 5.2a	2.22 ± 0.16A
SP+F								
90 day								
C	1.50 ± 0.23c	1.04 ± 0.18c	2.55 ± 0.40b	1.54 ± 0.12bc	1.46 ± 0.03AB	1.63 ± 0.14d	161.0 ± 6.4a	1.95 ± 0.03A
Mn_2_O_3_-NPs	2.12 ± 0.06ab	1.58 ± 0.08ab	3.69 ± 0.14a	1.81 ± 0.06a	1.34 ± 0.03AB	2.04 ± 0.01ab	119.2 ± 13.2b	2.35 ± 0.08A
Mn_2_O_3_	2.28 ± 0.02a	1.78 ± 0.06a	4.06 ± 0.08a	1.81 ± 0.05a	1.28 ± 0.03AB	2.24 ± 0.01a	104.3 ± 7.5b	1.92 ± 0.35A
MnSO_4_	1.80 ± 0.05bc	1.23 ± 0.07c	3.03 ± 0.11b	1.73 ± 0.02ab	1.47 ± 0.04A	1.76 ± 0.09cd	163.6 ± 6.0a	2.26 ± 0.01A
MnCl_2_	1.64 ± 0.07c	1.31 ± 0.06bc	2.96 ± 0.01b	1.50 ± 0.04c	1.26 ± 0.11B	1.98 ± 0.05bc	110.2 ± 22.4b	2.02 ± 0.05A
120 day								
C	1.45 ± 0.04A	0.79 ± 0.03B	2.23 ± 0.06B	1.41 ± 0.04B	1.84 ± 0.06A	1.58 ± 0.01ab	364.0 ± 53.0ab	1.69 ± 0.10c
Mn_2_O_3_-NPs	1.51 ± 0.07A	1.03 ± 0.04AB	2.54 ± 0.07AB	1.86 ± 0.04A	1.47 ± 0.11B	1.36 ± 0.01c	294.0 ± 49.0b	2.01 ± 0.05a
Mn_2_O_3_	1.65 ± 0.05A	1.26 ± 0.16A	2.92 ± 0.19A	1.79 ± 0.12AB	1.35 ± 0.15B	1.63 ± 0.06a	370.7 ± 63.8ab	1.66 ± 0.03c
MnSO_4_	1.51 ± 0.13A	1.08 ± 0.03AB	2.59 ± 0.14AB	1.84 ± 0.04A	1.39 ± 0.11B	1.41 ± 0.07bc	522.7 ± 43.2a	1.95 ± 0.08ab
MnCl_2_	1.40 ± 0.13A	0.98 ± 0.13AB	2.38 ± 0.24B	1.59 ± 0.23AB	1.46 ± 0.10B	1.52 ± 0.08abc	254.7 ± 27.5b	1.71 ± 0.11bc

Data are expressed as mean+standard error. Different letters show significant differences between treatments. Capital letters represent no statistical difference among treatments.

Total phenolic content was significantly altered by all three factors (i.e., application, Mn form and developmental stage) (**Table 2**). Briefly, the total phenolic content increased along with progressing the plant growth. Regarding SP condition, at the early (60 days) and mid (90 days) stages, the total phenolic content decreased by Mn treatments (except for MnSO_4_) compared with control. However, the reduced trend was only observed in Mn_2_O_3_ treatment at 120 days, possibly due to its early developmental phase raising from late germination. Moreover, foliar application of Mn stimulated the synthesis of phenolic compounds mainly in MnSO_4_ treatments (30%) compared to control and also its relevant treatment in SP condition (37%). However, MnCl_2_, other ionic forms of Mn, decreased the total phenolic content by 30% compared to the control. Therefore, the highest content was recorded by foliar application of MnSO_4_ at 120 days (**Table 2**). Similarly, total flavonoid content was differentially increased over time by treatments and applications as well. The data showed that the highest amount of total flavonoid is related to 90 day plants grown under SP conditions (**Table 2**). However, there were no remarkable differences between treatments (i.e., Mn forms and control). Notably, it is reasonable to infer that the synthesis of photosynthetic pigments and flavonoids might be slightly decreased during the flowering stage. Overall, despite not being significant, Mn_2_O_3_-NPs and MnSO_4_ appear to have more influence on flavonoid content (**Table 2**).

### 3.5 Heatmap and principal component analysis analyses

**Figure 5:**
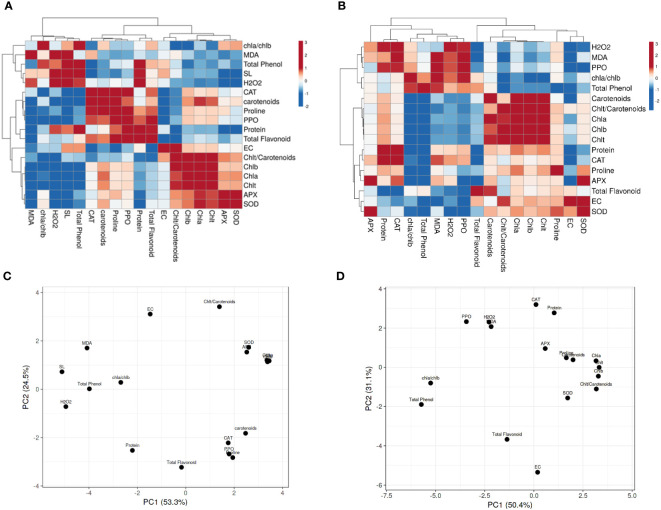
Heatmap and PCA analysis of Pearson’s correlation for the targeted parameters in 90 days’ plants in both SP **(A, C)** and SP+F **(B, D)** conditions. Pink and blue color represents positive and negative correlations, respectively.

The Pearson’s correlation was performed on all targeted parameters, and outputs were analyzed using a heatmap (**Figures 5**, **3S**). The heatmap related to 90 day’s plants in SP (**Figure 5A**) and SP+F (**Figure 5C**) conditions showed that each application has specific responses based on the correlation between parameters. In detail, a positive correlation was somehow found between oxidative enzymes activity, protein content and proline accumulation as well as with photosynthetic pigments and total phenolic content in SP condition, but not with H_2_O_2_ and MDA content. However, in SP+F condition, both stress indicators and oxidative enzyme activity were positively correlated. The PCA plot for 90 days’ of plants grown in SP condition accounted for 53.3% and 24.5% of the variance of PC1 and PC2 (**Figure 5B**), respectively, whereas the values of 50.4% and 32.1% were recorded for SP+F condition (**Figure 5D**). The PCA results also confirmed that the two applications impose some relatively different responses, but not entirely distinctive, in plants. This kind of difference was also observed in 60 and 120 days’ of plants (**Figure 3S**). Interestingly, H_2_O_2_ and MDA were negatively correlated with almost all other parameters at 60 days’ of plants, highlighting their competition to metabolize plant balance (**Figures 3S, A**). However, at the late developmental stage, the correlation coefficients were not highly significant, probably indicating plant adaptation status (**Figures 3S; B, C**). The relationships between growth, physiological and biochemical variables based on PCA plots (**Figure 3S; D-F**) were application and also developmental stage-dependent.

## 4 Discussion

The use of metal NPs has revolutionized the agricultural sector to improve the production of crop products. This event is done to find more effective and eco-friendly stimulators contributing to a more sustainable future (Pokrajac et al., 2021). However, the effectiveness of NPs in terms of being positive, safe and economically efficient compared to other counterparts is still an open issue in the nanotechnology discipline. Here, we used two application methods (SP and SP+F) to investigate the effect of Mn_2_O_3_-NPs on *A. annua* performance and compare it with their bulk and ionic forms during short- to long-term intervals from the germination stage to maturation. Seed germination is one of the most vulnerable processes in plant’s life cycle, supporting healthy and robust seedling development, which is largely affected by internal and external clues. Additionally, low crop productivity has been found to be linked with uneven seed germination. Research has proved that seed priming, a well-established method, can improve the quality of seeds and triggers seed germination efficiency and further plant growth (Adhikari et al., 2022; Thakur et al., 2022).

The findings presented in this study showed that SP with different Mn forms significantly alters the assayed attributes of germination and seedling growth (**Table 1**). Among Mn treatments, MnSO_4_ and Mn_2_O_3,_ respectively, improved and declined the germination-related parameters compared to the control. Mn_2_O_3_-NPs at the same concentration had a mild improving effect not as much different from control. Previous researches have reported that nano-priming significantly improves germination compared to ionic and hydropriming, mainly through accelerating water and nutrient uptake mediated by creating nanopores in seed coats (Mahakham et al., 2017; do Espirito Santo Pereira et al., 2021; Nile et al., 2022). Based on our results, however, this hypothesis was not proved for Mn_2_O_3_-NPs compared to MnSO_4_. The plant length and dry mass of seedlings grown at seed priming conditions also demonstrated the effectiveness of Mn as MnSO_4_>Mn_2_O_3_-NPs≥control>MnCl_2_>Mn_2_O_3_.

It has been proved that hydropriming is highly cost-effective and environment friendly to better the emergence and establishment of seedlings (Thakur et al., 2022). However, our results showed that priming with suitable ionic and nano forms of Mn can remarkably prompt these processes. Previous studies have reported the effectiveness of nutripriming with MnSO_4_ to accelerate germination rate (Munawar et al., 2013; Alejandro et al., 2020). On the other hand, a recent study showed that SP with MnO-NPs had comparatively less phytotoxicity than its bulk counterparts in watermelon seedlings (Kasote et al., 2021), which is in line with our results that nano forms of Mn are safer than their bulk. The mechanisms underlying the SP-induced modifications include the proper DNA conformation and reparation in embryos which is resulted in genome and protein integrity and, therefore, proceeding cell division (Thakur et al., 2022). In agreement, it is generally accepted that DNA polymerase conformational activation is mediated by a metal-based mechanism (Kirby et al., 2012), which can probably explain the Mn-induced germination.

The early responses of priming with Mn species were analyzed by germination-related parameters and the overall output confirms the critical role of seed-priming strategies (Devika et al., 2021). To further clarify the long-term responses of Mn-priming, the seedlings were grown for 120 days and then plant growth, physio-biochemical attributes were analyzed during this time. The plant growth at all tested times (60, 90, and 120, which are equal to specific developmental stages) followed the same trend as germination.

In other words, the higher growth rate in plants grown from seeds primed with MnSO_4_, control, and Mn_2_O_3_-NPs can be ascribed to the early and faster germination by these treatments, highlighting the importance of seed germination and priming strategy for successful establishment and higher productivity of germinated seedlings (Carrera-Castaño et al., 2020). In the present experiment, no obvious toxic symptoms of plants such as leaf wilt, necrosis, and yellowish leaves was observed, even in Mn_2_O_3_ treatment, which caused germination delay and subsequently lower plant growth. Interestingly, when we applied Mn treatments through foliar spray, all treatments except for Mn_2_O_3_ increased the plant growth compared to seed priming only, indicating the higher efficiency of SP+F combination to improve plant productivity. In confirmation of this statement, the results showed that foliar application of MnCl_2_ compensates the weak growth of seedlings developed from priming method. Foliar application of exogenous nutrients acts as a viable strategy to minimize nutrient efficiency in plants through fast delivery at the point of assimilation and therefore allows plant biofortification (Alshaal and El-Ramady, 2017; Dass et al., 2022).

Different processes involved in plant’s life cycle, like photosynthesis, ROS scavenging, and signaling pathway, have been found to be Mn-dependent (Alejandro et al., 2020). The results presented here also showed that Mn-induced changes are mainly reflected in the synthesis of photosynthetic pigments, phenolic compounds and some specific antioxidant enzymes. For example, photosynthetic pigments were generally increased under Mn treatments, especially in SP conditions. It’s worth mentioning that, it is difficult to determine which Mn form has the most influence on photosynthetic pigments since, at each time, different behavior was observed. However, it was reported that the optimum application of exogenous Mn can enhance photosynthetic efficiency under normal and stress conditions (Ahmad et al., 2019). Additionally, the Chl a/Chl b and total Chl/carotenoids ratios are considered to be vital parameters for photosynthesis efficiency (Jiménez-Lao et al., 2021). In the present study, an overall increased ratio of these parameters suggests a mild promotion of photosynthetic synthesis under Mn treatments. This could be due to improving the energy generation and regeneration of Ribulose-1,5-Bisphosphate (RuBP) involved in Calvin cycle. The total phenolic content was also significantly affected by either Mn treatments (in particular MnSO_4_) or application methods, highlighting the importance of Mn in phenylpropanoid pathways (Liu et al., 2019). Mn has been shown to serve as cofactor of the phenylalanine ammonia-lyase which is an important enzyme in the phenylpropanoid pathway to synthesize phenolic metabolites (Alejandro et al., 2020).

In accordance with our study, a recent study also showed that photosynthetic pigments, phenolic compounds and antioxidant profiles of watermelon seedlings were significantly affected by seed priming with 20 mg L^-1^MnO-NPs (Kasote et al., 2021). However, there was no clear trend of changes regarding the antioxidant flavonoid content as it increased in some treatments but no changes in some others compared to the control. The foliar-sprayed MnSO_4_ enhanced flavonoid content, which is in line with the results of Chen et al. (Li et al., 2020), reporting the increased flavonoid content in *Vitis vinifera* L treated by MnSO_4_. Despite not being significant in some cases, the overall decline in H_2_O_2_ and MDA content under Mn treatments was observed, which indicates the key role of Mn in ROS-scavenger-induced compounds (Alejandro et al., 2020). This was also in line with generally increased SOD activity, which reflects the protective nature of Mn pre-treatments, specifically by Mn bulk and ionic (MnCl_2_) forms. Increase SOD activity by metals such as Mn, Fe, and Zn has previously been reported, highlighting its key role as a cofactor of this enzyme (De Cuyper et al., 2017; Hu and Jinn, 2022). Declined oxidative markers, along with enhanced SOD activity, in this study, are mainly attributed to adaptive mechanism rather than defense system mechanism. Regarding other antioxidant enzymes, no clear trend of changes was found. However, an overall increase in PPO and APX activities was observed for MnSO_4_ treatments. Considering that no adverse symptoms were observed in MnSO_4_ treatments, up-regulated. Take this into account, the activation of antioxidant enzymes has been found to depend on Mn type, application method and finally, enzyme type, highlighting a diverse array of enzymatic responses exhibited by PCA analyses.

Antioxidant enzymes have been mainly reported to be the first line of defensive and protective mechanisms against any environmental factor such as abiotic stresses (Lei et al., 2022; Mittler et al., 2022; Najafi-Kakavand et al., 2022; Rahman et al., 2022; Raza et al., 2022a; Raza et al., 2022b; Shahid et al., 2022; Shaukat et al., 2022; Bhardwaj et al., 2023). At first, the superoxide radicals are converted to H_2_O_2_ by SOD activity which can be further decomposed into H_2_O by CAT and peroxidases (Hasanuzzaman et al., 2020). In addition, proline was significantly increased in almost all Mn treatments, especially by foliar-sprayed Mn_2_O_3_-NPs. It has been accepted that proline, a non-essential amino acid, plays a vital role in the maintenance of cellular redox homeostasis and cell’s energy status (Ghosh et al., 2022). Overall, the application of Mn treatments, except for its bulk form *via* both SP and foliar spray, improved *A. annua* performance by reducing H_2_O_2_ and MDA content. Mn is thought to scavenge free radicals itself or by increasing antioxidant enzyme activities, especially SOD and proline. Therefore, speaking generally, Mn as a micronutrient is involved in the scavenging of free radicals directly by itself or indirectly by activating various enzymes, top of them SOD (a primary antioxidative enzyme), and proline (Schmidt and Husted, 2019; Alejandro et al., 2020). On the other hand, different alteration of enzymes indicates the activation of specific antioxidant mechanisms by various forms of Mn, individually. Moreover, foliar application of Mn was only effective for a limited period after exposure since Mn is very little mobile in the plant and does not remobilize along plant organs. Considering the tested parameters, it is observed that the trend of changes was much more dynamic and significant at 90 days than 120 days, which also shows the adaptive status of plants at 120 days.

To sum up, comparing the effects of Mn_2_O_3_-NPs on *A.annua* performance in terms of morphological, and physio-biochemical attributes, our findings showed that NP form doesn’t have a remarkable priority to induce distinctive effects on plants performance compared to their conventional forms and control. In other words, if one of the Mn forms is supposed to be used to improve plant growth, based on the results, MnSO_4_ would be the best option.

## 5 Conclusion

**Figure 6:**
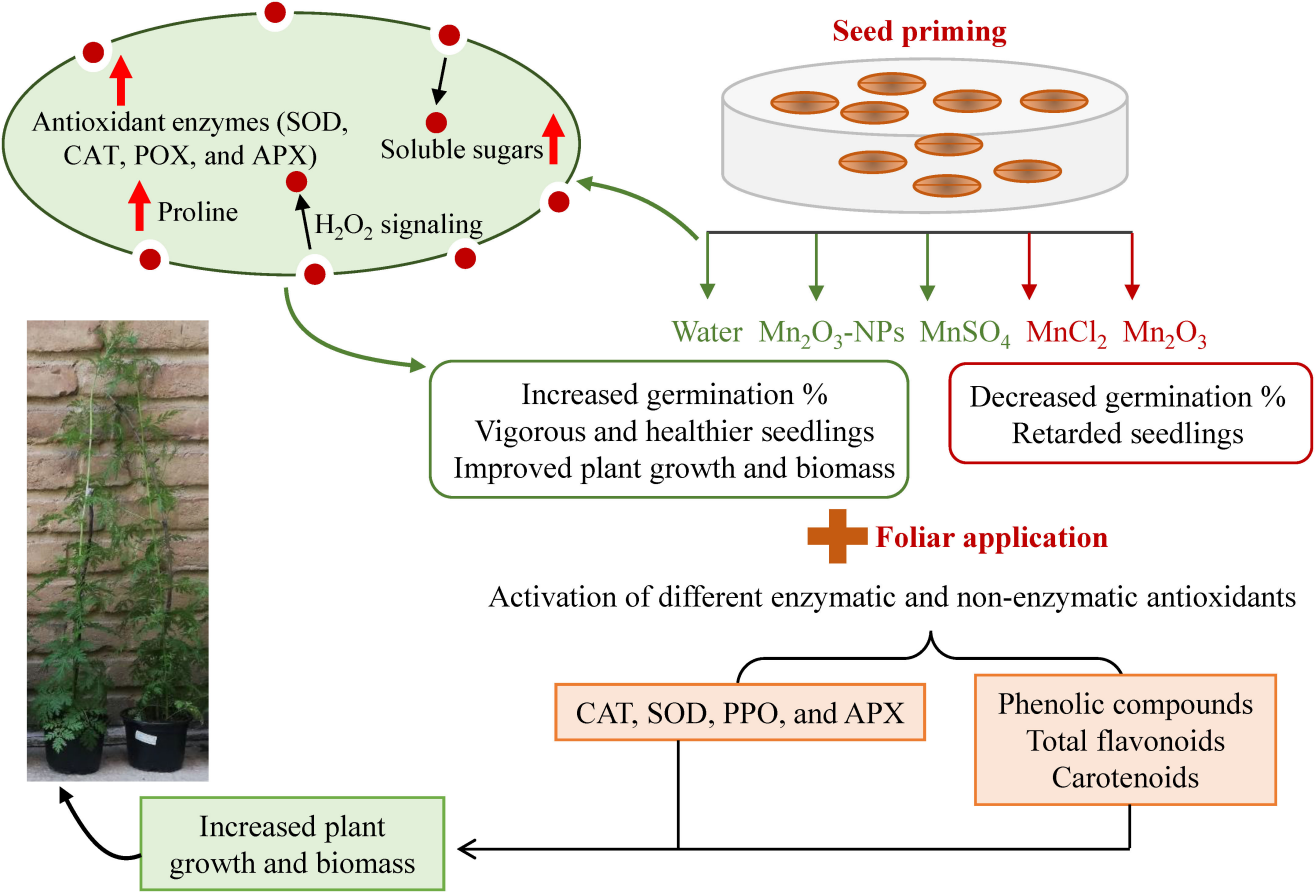
A schematic overview of the possible mechanism underlying improved plant growth and biomass based on different Mn application.

Our study showed that SP with different Mn forms could improve seed germination attributes and further seedling development of *A. annua.* Among treatments, priming with MnSO_4_, Mn_2_O_3_-NPs and water, respectively, resulted in the highest germination index and shortened the germination period. Similarly, vigorous and healthier seedlings were also observed in the mentioned treatments. On the other hand, foliar application of the mentioned Mn treatments helped in plant growth improvement even more. For instance, although SP with MnCl_2_ decreased germination and early growth, its foliar application compensated the retarded seedlings, indicating the difference between the two applications. Although plant growth did not change in some of the Mn treatments (for example, Mn_2_O_3_-NPs and MnCl_2_) compared to control, they all had their specific trend of changes in tested attributes. The biochemical analyses also suggest that each Mn form can activate different enzymatic and non-enzymatic antioxidants (**Figure 6**). However, the activation of SOD and proline can be considered a common Mn-induced response. After considering the overall attributes, including germination, morphological and physio-biochemical status, MnSO_4_ is highly recommended to be used in triggering plant growth during the early phase and therefore is cost- and resource-effective. However, other forms, such as MnCl_2_ and Mn_2_O_3_, which have more effect on the antioxidant defense systems, might be suitable under stress conditions. In addition, NPs, because of releasing more Mn^+2^ ions, are applicable to be used in very small amounts. Overall, the exogenous application of specific Mn as combined seed and foliar pre-treatments is recommended to first accelerate germination and then improve further plant growth by modulating antioxidant enzymes. Future studies regarding the stimulating application should explore the optimal dose of Mn_2_O_3_-NPs at a very low amount and investigate their real efficiency to be cost-effective.

## Data availability statement

The original contributions presented in the study are included in the article/**Supplementary Material**. Further inquiries can be directed to the corresponding author.

## Author contributions

HS conceived the idea and conducted the formal analysis, data curation, writing- review and editing. ACR supervised the research work. HS, AR, ACR, ID, and PVVP proofread and edited the manuscript. All authors have read and approved the final version of the manuscript.

## References

[B1] Adhikari B. Olorunwa O. J. Barickman T. C. (2022). Seed priming enhances seed germination and morphological traits of lactuca sativa l. under salt stress. Seeds 1 (2), 74–86. doi: 10.3390/seeds1020007

[B2] Ahmad I. Kamran M. Yang X. Meng X. Ali S. Ahmad S.. (2019). Effects of applying uniconazole alone or combined with manganese on the photosynthetic efficiency, antioxidant defense system, and yield in wheat in semiarid regions. Agric. Water Manage. 216, 400–414. doi: 10.1016/j.agwat.2019.02.025

[B3] Ahmad B. Shabbir A. Jaleel H. Khan M. M. A. Sadiq Y. (2018). Efficacy of titanium dioxide nanoparticles in modulating photosynthesis, peltate glandular trichomes and essential oil production and quality in mentha piperita l. Curr. Plant Biol. 13, 6–15. doi: 10.1016/j.cpb.2018.04.002

[B4] Ahmar S. Mahmood T. Fiaz S. Mora-Poblete F. Shafique M. S. Chattha M. S.. (2021). Advantage of nanotechnology-based genome editing system and its application in crop improvement. Front. Plant Sci. 12. doi: 10.3389/fpls.2021.663849 PMC8194497 34122485

[B5] Alejandro S. Höller S. Meier B. Peiter E. (2020). Manganese in plants: from acquisition to subcellular allocation. Front. Plant Sci. 11, 300. doi: 10.3389/fpls.2020.00300 32273877 PMC7113377

[B6] Alexieva V. Sergiev I. Mapelli S. Karanov E. (2001). The effect of drought and ultraviolet radiation on growth and stress markers in pea and wheat. Plant Cell Environ. 24 (12), 1337–1344. doi: 10.1046/j.1365-3040.2001.00778.x

[B7] Alshaal T. El-Ramady H. (2017). Foliar application: from plant nutrition to biofortification. Environment Biodiversity Soil Secur. 1 (2017), 71–83. doi: 10.21608/JENVBS.2017.1089.1006

[B8] Arnon D. I. (1949). Copper enzymes in isolated chloroplasts. polyphenoloxidase in beta vulgaris. Plant Physiol. 24 (1), 1. doi: 10.1104/pp.24.1.1 16654194 PMC437905

[B9] Bates L. S. Waldren R. P. Teare I. (1973). Rapid determination of free proline for water-stress studies. Plant Soil 39 (1), 205–207. doi: 10.1007/BF00018060

[B10] Beers R. F. Sizer I. W. (1952). A spectrophotometric method for measuring the breakdown of hydrogen peroxide by catalase. J. Biol. Chem. 195 (1), 133–140. doi: 10.1016/S0021-9258(19)50881-X 14938361

[B11] Bhardwaj S. Verma T. Raza A. Kapoor D. (2023). Silicon and nitric oxide-mediated regulation of growth attributes, metabolites and antioxidant defense system of radish (*Raphanus sativus* l.) under arsenic stress. Phyton-International J. Exp. Bot. 92 (3), 763–782. doi: 10.32604/phyton.2023.025672

[B12] Carrera-Castaño G. Calleja-Cabrera J. Pernas M. Gómez L. Oñate-Sánchez L. (2020). An updated overview on the regulation of seed germination. Plants 9 (6), 703. doi: 10.3390/plants9060703 32492790 PMC7356954

[B13] Chun O. K. Kim D.-O. Lee C. Y. (2003). Superoxide radical scavenging activity of the major polyphenols in fresh plums. J. Agric. Food Chem. 51 (27), 8067–8072. doi: 10.1021/jf034740d 14690398

[B14] Das M. Saxena N. Dwivedi P. D. (2009). Emerging trends of nanoparticles application in food technology: Safety paradigms. Nanotoxicology 3 (1), 10–18. doi: 10.1080/17435390802504237

[B15] Dass A. Rajanna G. A. Babu S. Lal S. K. Choudhary A. K. Singh R.. (2022). Foliar application of macro-and micronutrients improves the productivity, economic returns, and resource-use efficiency of soybean in a semiarid climate. Sustainability 14 (10), 5825. doi: 10.3390/su14105825

[B16] De Cuyper C. Struk S. Braem L. Gevaert K. De Jaeger G. Goormachtig S. (2017). Strigolactones, karrikins and beyond. Plant Cell Environ. 40 (9), 1691–1703. doi: 10.1111/pce.12996 28558130

[B17] Devika O. S. Singh S. Sarkar D. Barnwal P. Suman J. Rakshit A. (2021). Seed priming: a potential supplement in integrated resource management under fragile intensive ecosystems. Front. Sustain. Food Syst. 209. doi: 10.3389/fsufs.2021.654001

[B18] do Espirito Santo Pereira A. Caixeta Oliveira H. Fernandes Fraceto L. Santaella C. (2021). Nanotechnology potential in seed priming for sustainable agriculture. Nanomaterials 11 (2), 267. doi: 10.3390/nano11020267 33498531 PMC7909549

[B19] Dogan K. Erol E. Didem Orhan M. Degirmenci Z. Kan T. Gungor A.. (2022). Instant determination of the artemisinin from various artemisia annua l. extracts by LC-ESI-MS/MS and their in-silico modelling and *in vitro* antiviral activity studies against SARS-CoV-2. Phytochemical Anal. 33 (2), 303–319. doi: 10.1002/pca.3088 PMC8662158 34585460

[B20] Du W. Tan W. Yin Y. Ji R. Peralta-Videa J. R. Guo H.. (2018). Differential effects of copper nanoparticles/microparticles in agronomic and physiological parameters of oregano (Origanum vulgare). Sci. Total Environ. 618, 306–312. doi: 10.1016/j.scitotenv.2017.11.042 29131998

[B21] Ghosh U. Islam M. Siddiqui M. Cao X. Khan M. (2022). Proline, a multifaceted signalling molecule in plant responses to abiotic stress: understanding the physiological mechanisms. Plant Biol. 24 (2), 227–239. doi: 10.1111/plb.13363 34796604

[B22] Hadi Soltanabad M. Bagherieh-Najjar M. B. Mianabadi M. (2020). Carnosic acid content increased by silver nanoparticle treatment in rosemary (Rosmarinus officinalis l.). Appl. Biochem. Biotechnol. 191 (2), 482–495. doi: 10.1007/s12010-019-03193-w 31797151

[B23] Hasanuzzaman M. Bhuyan M. Zulfiqar F. Raza A. Mohsin S. M. Mahmud J. A.. (2020). Reactive oxygen species and antioxidant defense in plants under abiotic stress: Revisiting the crucial role of a universal defense regulator. Antioxidants 9 (8), 681. doi: 10.3390/antiox9080681 32751256 PMC7465626

[B24] Hu S.-H. Jinn T.-L. (2022). Impacts of Mn, fe, and oxidative stressors on MnSOD activation by AtMTM1 and AtMTM2 in arabidopsis. Plants 11 (5), 619. doi: 10.3390/plants11050619 35270089 PMC8912514

[B25] Jebara S. Jebara M. Limam F. Aouani M. E. (2005). Changes in ascorbate peroxidase, catalase, guaiacol peroxidase and superoxide dismutase activities in common bean (Phaseolus vulgaris) nodules under salt stress. J. Plant Physiol. 162 (8), 929–936. doi: 10.1016/j.jplph.2004.10.005 16146319

[B26] Jeevanandam J. Barhoum A. Chan Y. S. Dufresne A. Danquah M. K. (2018). Review on nanoparticles and nanostructured materials: history, sources, toxicity and regulations. Beilstein J. Nanotech. 9 (1), 1050–1074. doi: 10.3762/bjnano.9.98 PMC5905289 29719757

[B27] Jiménez-Lao R. Garcia-Caparros P. Pérez-Saiz M. Llanderal A. Lao M. T. (2021). Monitoring optical tool to determine the chlorophyll concentration in ornamental plants. Agronomy 11 (11), 2197. doi: 10.3390/agronomy11112197

[B28] Kasote D. M. Lee J. H. Jayaprakasha G. K. Patil B. S. (2021). Manganese oxide nanoparticles as safer seed priming agent to improve chlorophyll and antioxidant profiles in watermelon seedlings. Nanomaterials 11 (4), 1016. doi: 10.3390/nano11041016 33921180 PMC8071577

[B29] Khan I. Saeed K. Khan I. (2019). Nanoparticles: Properties, applications and toxicities. Arabian J. Chem. 12 (7), 908–931. doi: 10.1016/j.arabjc.2017.05.011

[B30] Kirby T. W. DeRose E. F. Cavanaugh N. A. Beard W. A. Shock D. D. Mueller G. A.. (2012). Metal-induced DNA translocation leads to DNA polymerase conformational activation. Nucleic Acids Res. 40 (7), 2974–2983. doi: 10.1093/nar/gkr1218 22169953 PMC3326329

[B31] Kralova K. Jampilek J. (2021). Responses of medicinal and aromatic plants to engineered nanoparticles. Appl. Sci. 11 (4), 1813. doi: 10.3390/app11041813

[B32] Kumari S. Khanna R. R. Nazir F. Albaqami M. Chhillar H. Wahid I.. (2022). Bio-synthesized nanoparticles in developing plant abiotic stress resilience: A new boon for sustainable approach. Int. J. Mol. Sci. 23 (8), 4452. doi: 10.3390/ijms23084452 35457269 PMC9025213

[B33] Landa P. (2021). Positive effects of metallic nanoparticles on plants: Overview of involved mechanisms. Plant Physiol. Biochem. 161, 12–24. doi: 10.1016/j.plaphy.2021.01.039 33561657

[B34] Lei Y. He H. Raza A. Liu Z. Xiaoyu D. Guijuan W.. (2022). Exogenous melatonin confers cold tolerance in rapeseed (Brassica napus l.) seedlings by improving antioxidants and genes expression. Plant Signaling Behav. 17 (1), 2129289. doi: 10.1080/15592324.2022.2129289 PMC9553147 36205498

[B35] Li W. Nguyen K. H. Tran C. D. Watanabe Y. Tian C. Yin X.. (2020). Negative roles of strigolactone-related SMXL6, 7 and 8 proteins in drought resistance in arabidopsis. Biomolecules 10 (4), 607. doi: 10.3390/biom10040607 32295207 PMC7226073

[B36] Liu P. Huang R. Hu X. Jia Y. Li J. Luo J.. (2019). Physiological responses and proteomic changes reveal insights into stylosanthes response to manganese toxicity. BMC Plant Biol. 19 (1), 1–21. doi: 10.1186/s12870-019-1822-y 31113380 PMC6530018

[B37] Lutts S. Almansouri M. Kinet J.-M. (2004). Salinity and water stress have contrasting effects on the relationship between growth and cell viability during and after stress exposure in durum wheat callus. Plant Sci. 167 (1), 9–18. doi: 10.1016/j.plantsci.2004.02.014

[B38] Mahakham W. Sarmah A. K. Maensiri S. Theerakulpisut P. (2017). Nanopriming technology for enhancing germination and starch metabolism of aged rice seeds using phytosynthesized silver nanoparticles. Sci. Rep. 7 (1), 1–21. doi: 10.1038/s41598-017-08669-5 28811584 PMC5557806

[B39] Mittler R. Zandalinas S. I. Fichman Y. Van Breusegem F. (2022). Reactive oxygen species signalling in plant stress responses. Nat. Rev. Mol. Cell Biol. 1-17. doi: 10.1038/s41580-022-00499-2 35760900

[B40] Moazzami Farida S. H. Karamian R. Albrectsen B. R. (2020). Silver nanoparticle pollutants activate oxidative stress responses and rosmarinic acid accumulation in sage. Physiologia plantarum 170 (3), 415–432. doi: 10.1111/ppl.13172 32705693

[B41] Munawar M. Ikram M. Iqbal M. Raza M. M. Habib S. Hammad G.. (2013). Effect of seed priming with zinc, boron and manganese on seedling health in carrot (DaucuscarotaL.). Int. J. Agric. Crop Sci. 5 (22), 2697.

[B42] Najafi-Kakavand S. Karimi N. Ghasempour H.-R. Raza A. Chaichi M. Modarresi M. (2022). Role of jasmonic and salicylic acid on enzymatic changes in the root of two alyssum inflatum náyr. populations exposed to nickel toxicity. J. Plant Growth Regul., 1–18. doi: 10.1007/s00344-022-10648-8

[B43] Nile S. H. Thiruvengadam M. Wang Y. Samynathan R. Shariati M. A. Rebezov M.. (2022). Nano-priming as emerging seed priming technology for sustainable agriculture–recent developments and future perspectives. J. Nanobiotech. 20 (1), 1–31. doi: 10.1186/s12951-022-01423-8 PMC9164476 35659295

[B44] Nourozi E. Hosseini B. Maleki R. Abdollahi Mandoulakani B. (2019). Iron oxide nanoparticles: a novel elicitor to enhance anticancer flavonoid production and gene expression in dracocephalum kotschyi hairy-root cultures. J. Sci. Food Agric. 99 (14), 6418–6430. doi: 10.1002/jsfa.9921 31294466

[B45] Pérez-de-Luque A. (2017). Interaction of nanomaterials with plants: what do we need for real applications in agriculture? Front. Environ. Sci. 5, 12. doi: 10.3389/fenvs.2017.00012

[B46] Pokrajac L. Abbas A. Chrzanowski W. Dias G. M. Eggleton B. J. Maguire S.. (2021). Nanotechnology for a sustainable future: Addressing global challenges with the international network4sustainable nanotechnology (ACS Publications). doi: 10.1021/acsnano.1c10919 34910476

[B47] Pullagurala V. L. R. Adisa I. O. Rawat S. Kalagara S. Hernandez-Viezcas J. A. Peralta-Videa J. R.. (2018a). ZnO nanoparticles increase photosynthetic pigments and decrease lipid peroxidation in soil grown cilantro (Coriandrum sativum). Plant Physiol. Biochem. 132, 120–127. doi: 10.1016/j.plaphy.2018.08.037 30189415

[B48] Pullagurala V. L. R. Adisa I. O. Rawat S. Kim B. Barrios A. C. Medina-Velo I. A.. (2018b). Finding the conditions for the beneficial use of ZnO nanoparticles towards plants-a review. Environ. pollut. 241, 1175–1181. doi: 10.1016/j.envpol.2018.06.036 30029327

[B49] Rahman M. A. Woo J. H. Lee S.-H. Park H. S. Kabir A. H. Raza A.. (2022). Regulation of Na+/H+ exchangers, Na+/K+ transporters, and lignin biosynthesis genes, along with lignin accumulation, sodium extrusion, and antioxidant defense, confers salt tolerance in alfalfa. Front. Plant Sci. 13, 1041764–1041764. doi: 10.3389/fpls.2022.1041764 36420040 PMC9676661

[B50] Raymond J. Rakariyatham N. Azanza J. (1993). Purification and some properties of polyphenoloxidase from sunflower seeds. Phytochemistry 34 (4), 927–931. doi: 10.1016/S0031-9422(00)90689-7

[B51] Raza A. Charagh S. García-Caparrós P. Rahman M. A. Ogwugwa V. H. Saeed F.. (2022a). Melatonin-mediated temperature stress tolerance in plants. GM Crops Food 13 (1), 196–217. doi: 10.1080/21645698.2022.2106111 35983948 PMC9397135

[B52] Raza A. Salehi H. Rahman M. A. Zahid Z. Madadkar Haghjou M. Najafi-Kakavand S.. (2022b). Plant hormones and neurotransmitter interactions mediate antioxidant defenses under induced oxidative stress in plants. Front. Plant Sci. 13. doi: 10.3389/fpls.2022.961872 PMC9514553 36176673

[B53] Sabet H. Mortazaeinezhad F. (2018). Yield, growth and fe uptake of cumin (Cuminum cyminum l.) affected by fe-nano, fe-chelated and fe-siderophore fertilization in the calcareous soils. J. Trace Elements Med. Biol. 50, 154–160. doi: 10.1016/j.jtemb.2018.06.020 30262273

[B54] Salehi H. Chehregani A. Lucini L. Majd A. Gholami M. (2018). Morphological, proteomic and metabolomic insight into the effect of cerium dioxide nanoparticles to phaseolus vulgaris l. under soil or foliar application. Sci. Total Environ. 616, 1540–1551. doi: 10.1016/j.scitotenv.2017.10.159 29066204

[B55] Salehi H. De Diego N. Rad A. C. Benjamin J. J. Trevisan M. Lucini L. (2021a). Exogenous application of ZnO nanoparticles and ZnSO4 distinctly influence the metabolic response in phaseolus vulgaris l. Sci. Total Environ. 778, 146331. doi: 10.1016/j.scitotenv.2021.146331 33725605

[B56] Salehi H. Miras-Moreno B.a. Chehregani Rad A. Pii Y. Mimmo T. Cesco S.. (2019). Relatively low dosages of CeO2 nanoparticles in the solid medium induce adjustments in the secondary metabolism and ionomic balance of bean (Phaseolus vulgaris l.) roots and leaves. J. Agric. Food Chem. 68 (1), 67–76. doi: 10.1021/acs.jafc.9b05107 31710472

[B57] Salehi H. Rad A. C. Sharifan H. Raza A. Varshney R. K. (2021b). Aerially applied zinc oxide nanoparticle affects reproductive components and seed quality in fully grown bean plants (Phaseolus vulgaris l.). Front. Plant Sci. 12. doi: 10.3389/fpls.2021.808141 PMC8790032 35095979

[B58] Santiago E. F. Pontes M. S. Arruda G. J. Caires A. R. Colbeck I. Maldonado-Rodriguez R.. (2020). “Understanding the interaction of nanopesticides with plants,”. Nanopesticides Springer), 69–109. doi: 10.1007/978-3-030-44873-8_4

[B59] Schmidt S. B. Husted S. (2019). The biochemical properties of manganese in plants. Plants 8 (10), 381. doi: 10.3390/plants8100381 31569811 PMC6843630

[B60] Shahhoseini R. Azizi M. Asili J. Moshtaghi N. Samiei L. (2020). Effects of zinc oxide nanoelicitors on yield, secondary metabolites, zinc and iron absorption of feverfew (Tanacetum parthenium (L.) Schultz bip.). Acta Physiologiae plantarum 42 (4), 1–18. doi: 10.1007/s11738-020-03043-x

[B61] Shahid S. Kausar A. Zahra N. Hafeez M. B. Raza A. Ashraf M. Y. (2022). Methionine-induced regulation of secondary metabolites and antioxidants in maize (Zea mays l.) subjected to salinity stress. Gesunde Pflanzen, 1–13. doi: 10.3390/plants9121745

[B62] Shaukat K. Baksh G. Zahra N. Hafeez M. B. Raza A. Samad A.. (2022). Foliar application of thiourea, salicylic acid, and kinetin alleviate salinity stress in maize grown under etiolated and de-etiolated conditions. Discover Food 2 (1), 1–14. doi: 10.3390/plants9121745

[B63] Shen Q. Huang H. Xie L. Hao X. Kayani S. Liu H.. (2022). Basic helix-Loop-Helix transcription factors AabHLH2 and AabHLH3 function antagonistically with AaMYC2 and are negative regulators in artemisinin biosynthesis. Front. Plant Sci. 13. doi: 10.3389/fpls.2022.885622 PMC9207477 35734250

[B64] Sobarzo-Bernal O. Gómez-Merino F. C. Alcántar-González G. Saucedo-Veloz C. Trejo-Téllez L. I. (2021). Biostimulant effects of cerium on seed germination and initial growth of tomato seedlings. Agronomy 11 (8), 1525. doi: 10.3390/agronomy11081525

[B65] Stewart R. R. Bewley J. D. (1980). Lipid peroxidation associated with accelerated aging of soybean axes. Plant Physiol. 65 (2), 245–248. doi: 10.1104/pp.65.2.245 16661168 PMC440305

[B66] Szőllősi R. Molnár Á. Kondak S. Kolbert Z. (2020). Dual effect of nanomaterials on germination and seedling growth: Stimulation vs. phytotoxicity. Plants 9 (12), 1745. doi: 10.3390/plants9121745 33321844 PMC7763982

[B67] Talankova-Sereda T. Liapina K. Shkopinskij E. Ustinov A. Kovalyova A. Dulnev P.. (2016). “The influence of Cu и Co nanoparticles on growth characteristics and biochemical structure of mentha longifolia in vitro,” in Nanophysics, nanophotonics, surface studies, and applications (International Journal of Biosensors & Bioelectronics). : Springer), 427–436.

[B68] Thakur M. Tiwari S. Kataria S. Anand A. (2022). Recent advances in seed priming strategies for enhancing planting value of vegetable seeds. Scientia Hortic. 305, 111355. doi: 10.1016/j.scienta.2022.111355

[B69] Tian H. Ghorbanpour M. Kariman K. (2018). Manganese oxide nanoparticle-induced changes in growth, redox reactions and elicitation of antioxidant metabolites in deadly nightshade (Atropa belladonna l.). Ind. Crops Products 126, 403–414. doi: 10.1016/j.indcrop.2018.10.042

[B70] Velázquez-Gamboa M. C. Rodríguez-Hernández L. Abud-Archila M. Gutiérrez-Miceli F. A. González-Mendoza D. Valdez-Salas B.. (2021). Agronomic biofortification of stevia rebaudiana with zinc oxide (ZnO) phytonanoparticles and antioxidant compounds. Sugar Tech 23 (2), 453–460. doi: 10.1007/s12355-020-00897-w

[B71] Wu H. Li Z. (2021). Recent advances in nano-enabled agriculture for improving plant performance. Crop J 10(1)1–12. doi: 10.1016/j.cj.2021.06.002

[B72] Ye Y. Medina-Velo I. A. Cota-Ruiz K. Moreno-Olivas F. Gardea-Torresdey J. L. (2019). Can abiotic stresses in plants be alleviated by manganese nanoparticles or compounds? Ecotoxicol. Environ. Saf. 184, 109671. doi: 10.1016/j.ecoenv.2019.109671 31539809

[B73] Zahra W. Rai S. N. Birla H. Singh S. S. Rathore A. S. Dilnashin H.. (2020). “Economic importance of medicinal plants in Asian countries,” in Bioeconomy for sustainable development (Bioeconomy for Sustainable Development; Springer), 359–377. doi: 10.1007/978-981-13-9431-7_19

[B74] Zhishen J. Mengcheng T. Jianming W. (1999). The determination of flavonoid contents in mulberry and their scavenging effects on superoxide radicals. Food Chem. 64 (4), 555–559. doi: 10.1016/S0308-8146(98)00102-2

