# Supplementary material for: The comparative effects of manganese nanoparticles and their counterparts (bulk and ionic) in Artemisia annua plants via seed priming and foliar application
Source: Front Plant Sci. 2023 Jan 19;13:1098772. doi: 10.3389/fpls.2022.1098772 (PMC9893273; doi:10.3389/fpls.2022.1098772)
Supplement: Supplementary file 1 [file DataSheet_1.docx]

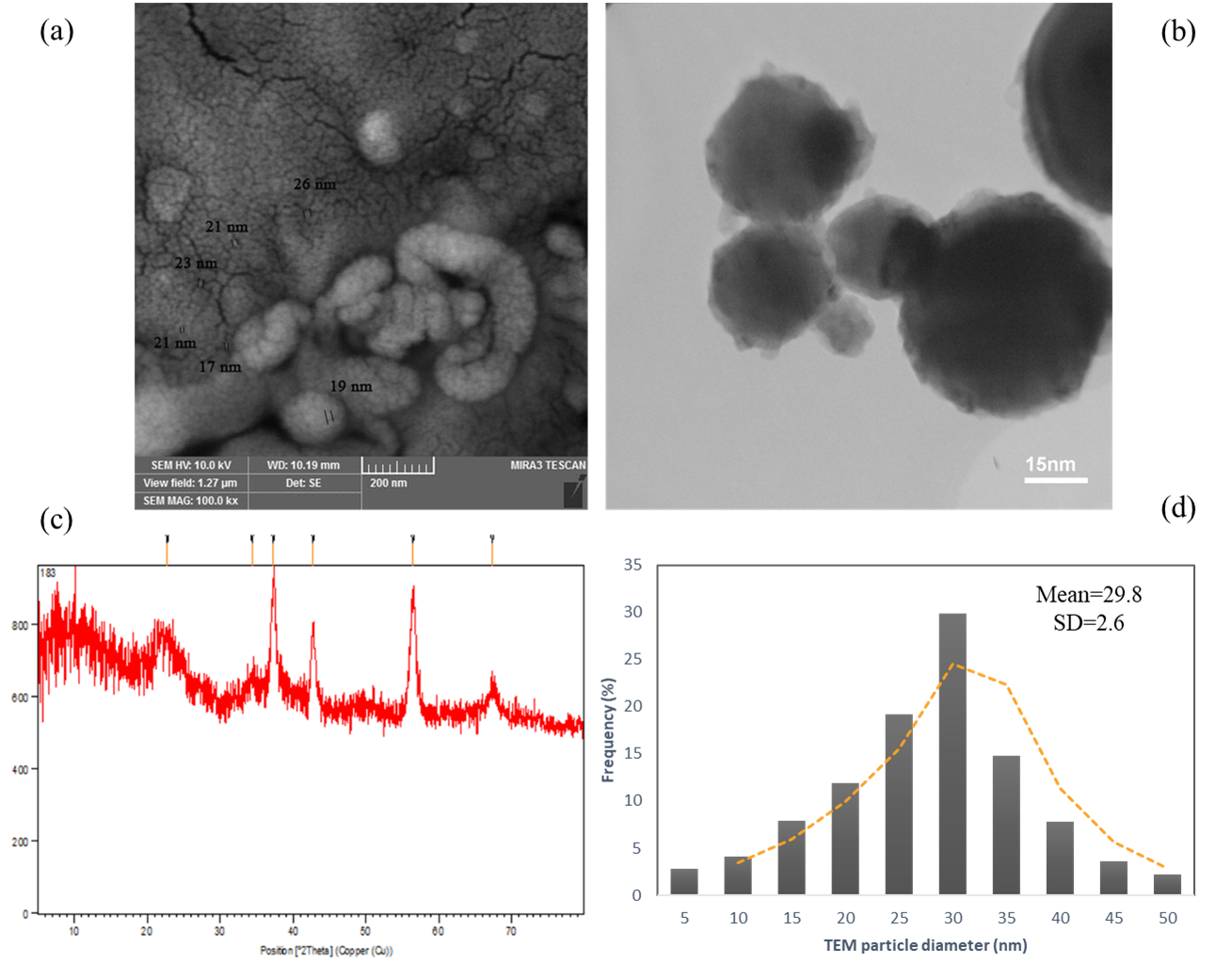


**Figure S1.** Mn_2_O_3_ NPs features including, scanning electron microscopy (SEM) (a), and transmission electron microscopy (TEM) (b) images, X-ray diffraction (XRD) (c), and size distribution (e).

**Figure S2.** Germination percentage of seeds primed with different dosages of Mn_2_O_3_-NPs at two different time exposure (3 and 6 hours) to assay NPs phytotoxicity.

| 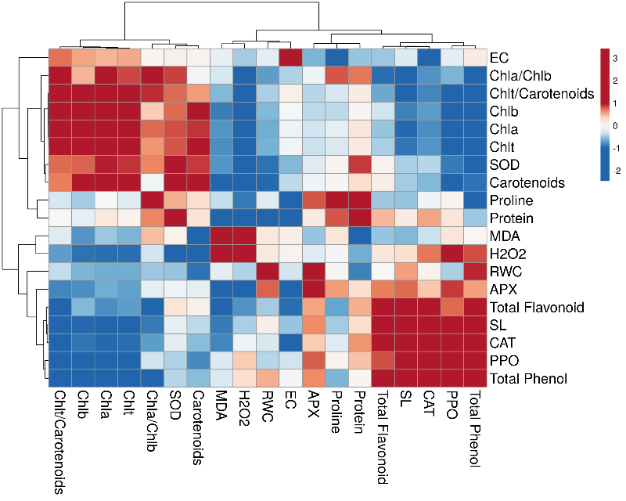  (a) | 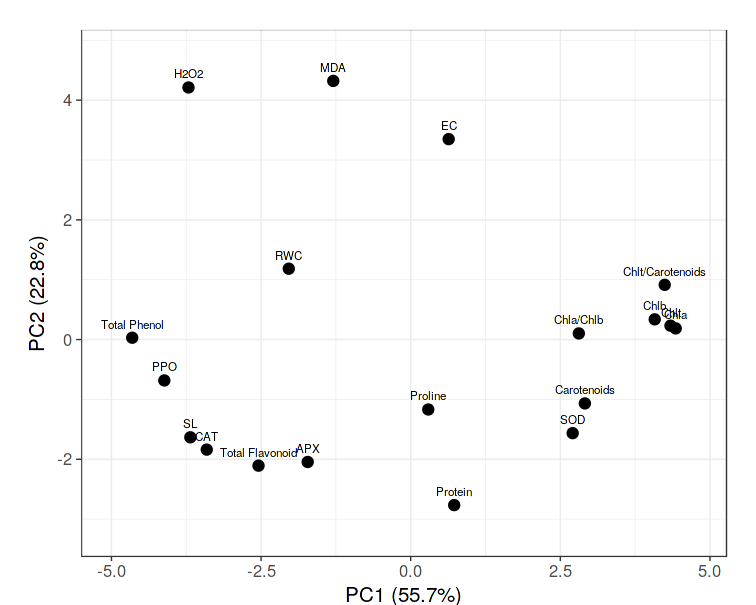  (d) |
| --- | --- |
| 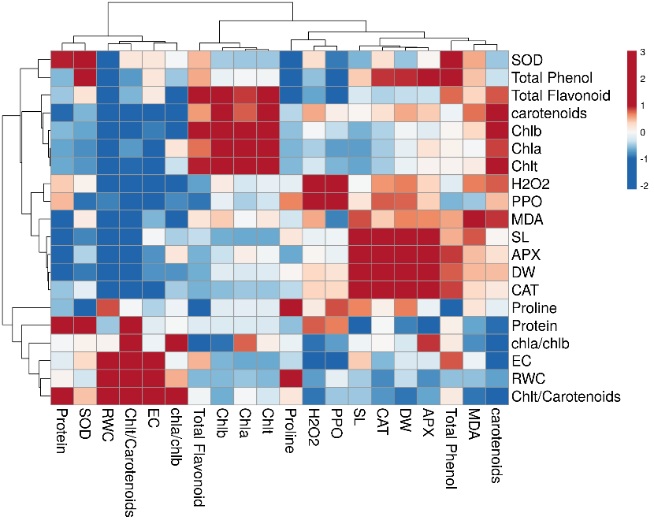  (b) | 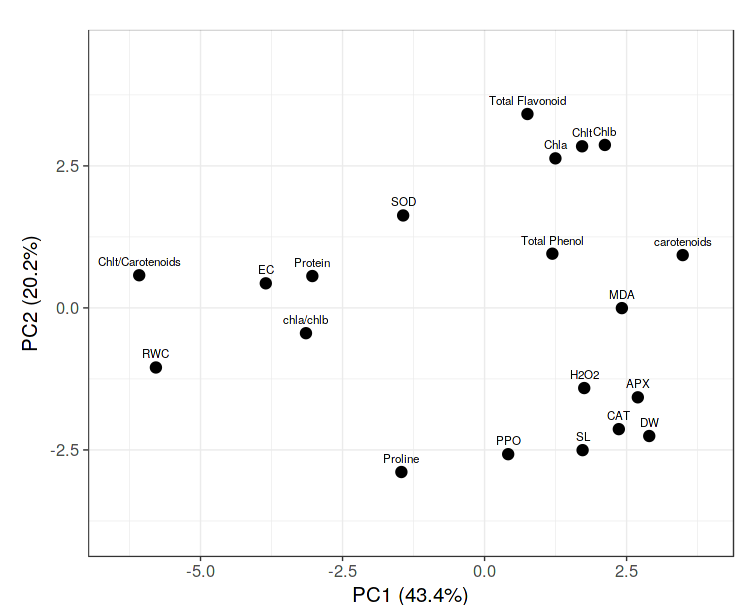  (e) |
| 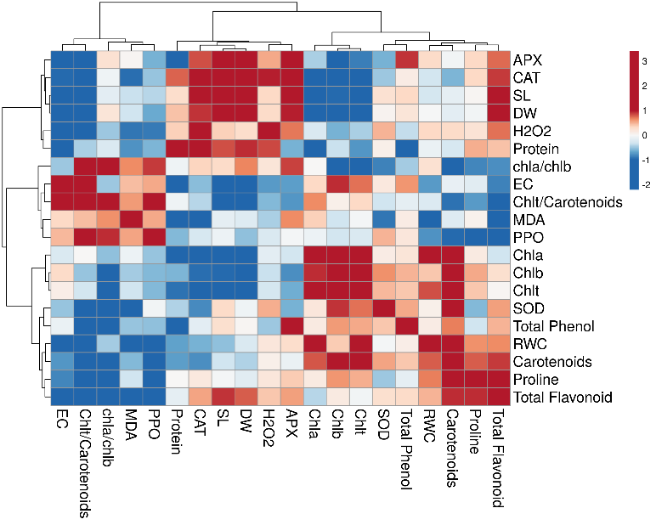  (c) | 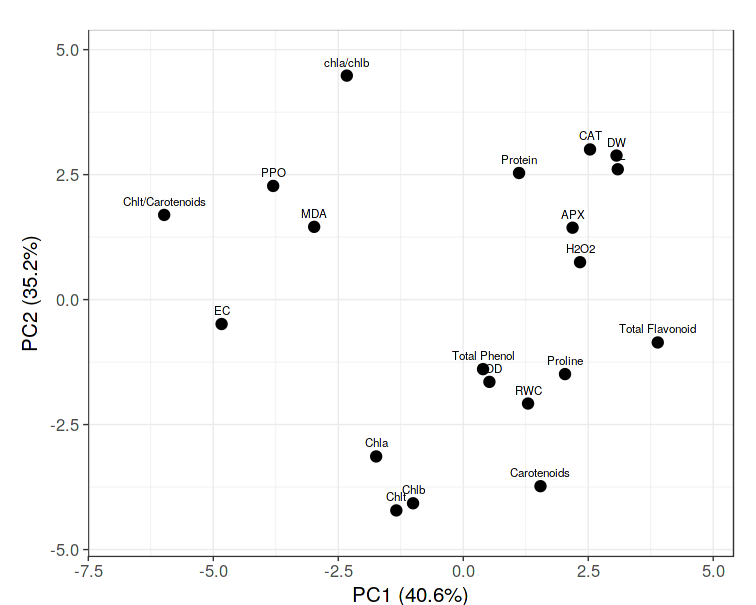  (f) |

**Figure S3.** Heatmap and PCA analyses of Pearson’s correlation for the targeted parameters in 60 days’ plants at SP condition (a, d), 120 days’ plants in both SP (b, e) and SP+F (c, f) conditions. Pink and blue color represents positive and negative correlations, respectively.
